# Preterm newborns at Kangaroo Mother Care: a cohort follow-up from birth to six months

**DOI:** 10.1590/0103-0582201432213113

**Published:** 2014-06

**Authors:** Maria Alexsandra da S. Menezes, Daniela Cavalcante Garcia, Enaldo Vieira de Melo, Rosana Cipolotti

**Affiliations:** 1 UFS, SE, Brasil

**Keywords:** Kangaroo-Mother Care Method, infant, premature, growth, breast feeding

## Abstract

**OBJECTIVE:**

To evaluate clinical outcomes, growth and exclusive breastfeeding rates in premature infants assisted by Kangaroo Mother Care at birth, at discharge and at six months of life.

**METHODS:**

Prospective study of a premature infants cohort assisted by Kangaroo Mother Care in a tertiary public maternity in Northeast Brazil with birth weight ≤1750g and with clinical conditions for Kangaroo care.

**RESULTS:**

The sample was composed by 137 premature infants, being 62.8% female, with average birth weight of 1365±283g, average gestational age of 32±3 weeks and 26.2% were adequate for gestational age. They have been admitted in the Kangaroo Ward with a median of 13 days of life, weighing 1430±167g and, at this time, 57.7% were classified as small for corrected gestational age. They were discharged with 36.8±21.8 days of chronological age, weighing 1780±165g and 67.9% were small for corrected gestational age. At six months of life (n=76), they had an average weight of 5954±971g, and 68.4% presented corrected weight for gestational age between percentiles 15 and 85 of the World Health Organization (WHO) weight curve. Exclusive breastfeeding rate at discharge was 56.2% and, at six months of life, 14.4%.

**CONCLUSIONS:**

In the studied sample, almost two thirds of the children assisted by Kangaroo Mother Care were, at six months of life, between percentiles 15 and 85 of the WHO weight curves. The frequency of exclusive breastfeeding at six months was low.

## Abstract (es)

**OBJETIVO:**

Evaluar la evolución clínica, el crecimiento y la tasa de lactancia materna exclusiva de recién nacidos prematuros asistidos por el Método Canguro, al nacimiento, en el alta y a los seis meses de edad.

**MÉTODOS:**

Estudio prospectivo de una cohorte de prematuros atendidos por el Método Canguro, en una maternidad pública de nivel terciario en el Nordeste de Brasil, con peso al nacer ≤1750g y en condiciones clínicas necesarias a la aplicación del método.

**RESULTADOS:**

La muestra se constituyó por 137 recién nacidos prematuros, siendo el 62,8% del sexo femenino, con peso mediano al nacer de 1365±283g, edad gestacional mediana de 32±3 semanas y 26,2% eran adecuados para la edad gestacional. Se admitieron en la Enfermería Canguro con mediana de 13 días de vida, pesando 1430±167g en promedio, siendo que, en ese momento, el 57,7% fueron clasificados como pequeño para la edad gestacional corregida. Tuvieron alta hospitalaria con 36,8±21,8 días de vida, pesando 1780±165g y el 67,9% eran pequeños para la edad gestacional corregida. A los seis meses de edad cronológica (n=76), tenían peso mediano de 5954±971g, estando el 68,4% con peso corregido para la edad gestacional entre los percentiles 15 y 85 de la curva de la Organización Mundial de la Salud (OMS). La frecuencia de lactancia materna exclusiva en el alta fue del 56,2% y, a los seis meses de edad cronológica, del 14,4%.

**CONCLUSIONES:**

En la muestra estudiada, aproximadamente dos tercios de los niños asistidos por el Método Canguro se encontraban, a los seis meses de edad cronológica, entre los percentiles 15 y 85 de la curva de peso corporal de OMS. La frecuencia de lactancia exclusiva a los seis meses fue baja.

## Introduction

There is an estimated annual rate of around 15 million births of preterm infants in the world, representing one in every 10 births. Many of the survivors will present learning, visual, and/or hearing problems, among others^(^ 1 ^)^. 

The Kangaroo Mother Care Program or Kangaroo Method (KM), originated in Colombia in 1978, includes the skin-to-skin contact between mother and newborn (NB) started as early as possible, having emerged in that country as an alternative to stimulate early hospital discharge in clinically stable low birth weight (LBW) infants^(^ 2 ^)^. In Brazil, this program's main goal is to encourage the formation of the bond between parents and babies, and it is a national health policy, launched by the Ministry of Health as Standards for Humanized Care to Low Weight Newborns - Kangaroo Method (SHCLWNB-KM), since 2000^(^ 3 ^)^.

The main benefits attributed to KM include: reduction of hypothermia, sepsis, length of hospital stay, and risk of mortality, at hospital discharge or with 40 weeks of corrected age^(^ 4 ^)^, besides positive impact on the cognitive and motor development of preterm infants^(^ 5 ^)^, maintenance of stability during transport of preterm or term infants^(^ 6 ^)^, as well as vital signs in physiological levels, even when performed in preterm infants under mechanical ventilation and hemodynamically stable^(^ 7 ^)^. The method avoids prolonged separation between mother and child, what could contribute for the insufficient production of milk, low affective bond, and increase of morbidities^(^ 8 ^)^, facilitating exclusive breastfeeding for LWNB until 6 months of life^(^ 9 ^)^, besides being related to a longer period of breastfeeding and higher production of milk^(^ 10 ^)^. In a recent systematic review published by Cochrane, the authors concluded that there is enough evidence to recommend the use of KM in stable LBW infants^(^ 4 ^)^. 

However, a study assessing 176 Brazilian maternity-hospitals, qualified between 2000 and 2003, shows that only 47.3% implemented the three stages of the method^(^ 11 ^)^. Véras and Traverso-Yépez^(^ 12 ^)^ state that the implementation policy of the KM does not value the social, cultural, and environmental aspects involved in motherhood. Other authors suggest that the success of the implantation of the KM depends on the training of health professionals and the facilities the hospital offers to mothers for its permanence. Furthermore, the state laws of implementation, such as those issued by the Brazilian Health Ministry, do not establish resources for the follow-up, the monitoring, and the assessment of the program. In this process, the professionals' resistance to family participation has given the KM a hospital character, with the extending length of rooming-in hospital stays and hospital discharge similar to conventional criteria, with inconsistencies between speech and practice^(^ 13 ^)^.

Considering these principles, the present study aimed to assess clinical progression, growth, and rate of exclusive breastfeeding of preterm infants assisted by the KM in a public institution of the Northeast region of Brazil in three moments: at birth, at hospital discharge, and at 6 months old. 

## Method

The present study used a prospective cohort, and was performed at a tertiary public maternity, in Northeastern Brazil. In this unit, there are about 4,400 deliveries per year, resulting in 31.2% LBW infants and approximately 8% very low birth weight (VLBW) newborns. The service has 36 hospital beds at the Neonatal Intensive Care Unit (NICU), 25 beds at the Intermediate Care Unit (ICU), 14 beds at the Kangaroo Ward, and 35 rooming-in beds, with mean annual occupancy rates of 121%. The occupancy rate of the NICU is frequently higher than the capacity of the service, so the service recourses to improvised beds. 

The study included preterm newborns (PTNB) born between July 1st, 2011 and January 31, 2012, with birth weight (BW) lower than or equal to 1,750g, who were in ambient air, without hydric venous support, weighing more than 1,250g and whose mother had agreed to participate in the KM. Those with congenital malformations that could interfere in the evolution of the patient were excluded. 

The following clinical intercurrences were researched during hospital stay: bronchopulmonary dysplasia (BPD), defined as the need for oxygen therapy for 28 days or more after birth; apnoea, defined as the interruption of air flow in the upper airways evolving to bradycardia and/or cyanosis; hypothermia, defined as axillar temperature <36.5°C, and need of third-line antibiotics (third generation cephalosporin or vancomycin, isolated, or in association).

Data were obtained from the analysis of the medical records; the interviews with mothers and the physical examination of the NB were always performed by the same evaluator. Some information regarding type of feeding, surgical correction of retinopathy of prematurity (ROP) after hospital discharge and need for new hospitalization were obtained also over the phone, when the consultation at 6 months old was not possible. To determine gestational age at birth, the New Ballard Score was applied^(^ 14 ^)^ and, to classify the weigh gain adequacy at birth, Alexander curves^(15) ^were applied, considering adequate for gestational age (AGA) those between the 10^th^ and the 90^th^ percentiles and small for gestational age (SGA) those below the 10^th^ percentile. To verify the weight adequacy to corrected age, Xavier curves^(16) ^were applied at admission to the Kangaroo Ward and at hospital discharge; at 6 months of chronological age; the curves of the World Health Organization (WHO) were applied according to corrected age^(^ 17 ^)^. 

The maternity hospital has its own protocol for the follow-up of preterm infants, but, for the study's data collection, an additional consultation was scheduled at 6 months of chronological age.

The evolution and assessment of weight adequacy in the PTNBs were performed in four different moments: T_0_, at birth; T_1_, at admission in the Kangaroo Ward; T_2_, at hospital discharge; and T_3_, at 6 months old.

The categorical variables were presented by means of frequency, as percentage, considering the confidence interval of 95%. The mean and standard deviation were calculated for the quantitative variables. In some cases, these variables were presented as median and percentiles. For the measures of associations between qualitative variables, the Fisher exact test and the chi-square test were used. Statistical analysis was performed by the Statistical Package for the Social Sciences (SPSS) software, version 13.0 for Windows.

The project was approved by the Research Ethics Committee of Universidade Federal de Sergipe.

## Results

In the studied period, 223 potentially eligible NBs were born, but 86 were excluded (41 due to death before admission at the Kangaroo Nursery, 41 due to non-adhesion to the method by the mother, one by maternal death, and three due to maternal refusal in participating), and the sample included 137 PTNBs.

The mean maternal age was 26±7 years, ranging from 13 to 44 years. Mother with up to 19 years remained 24.1% of the sample. Mothers who lived with the father of the child were 81.8%; 66% declared a monthly income lower than one minimum salary and one third declared some remunerated occupation; 60.5% were from the countryside of Sergipe or from other state; 44.5% attended four or less prenatal consultations; 41.6% were primiparous. Among the mothers of the babies assessed, 44.5% had a history of pregnancy-specific hypertensive disease, 36.5% of premature rupture of membranes (PRM), and 39% of infection of the urinary tract; 76.6% received at least one dose of antenatal corticosteroid, and 61.3% evolved to cesarean section.

Regarding the gender, 86 NBs (62.8%) were female. Multiple births was found in 15.3% of participants. Mean gestational age at birth was 32±3 weeks. Data on the conditions of birth and assistance to the PTNB in the delivery room are in Table 1. 

**Table 1:**
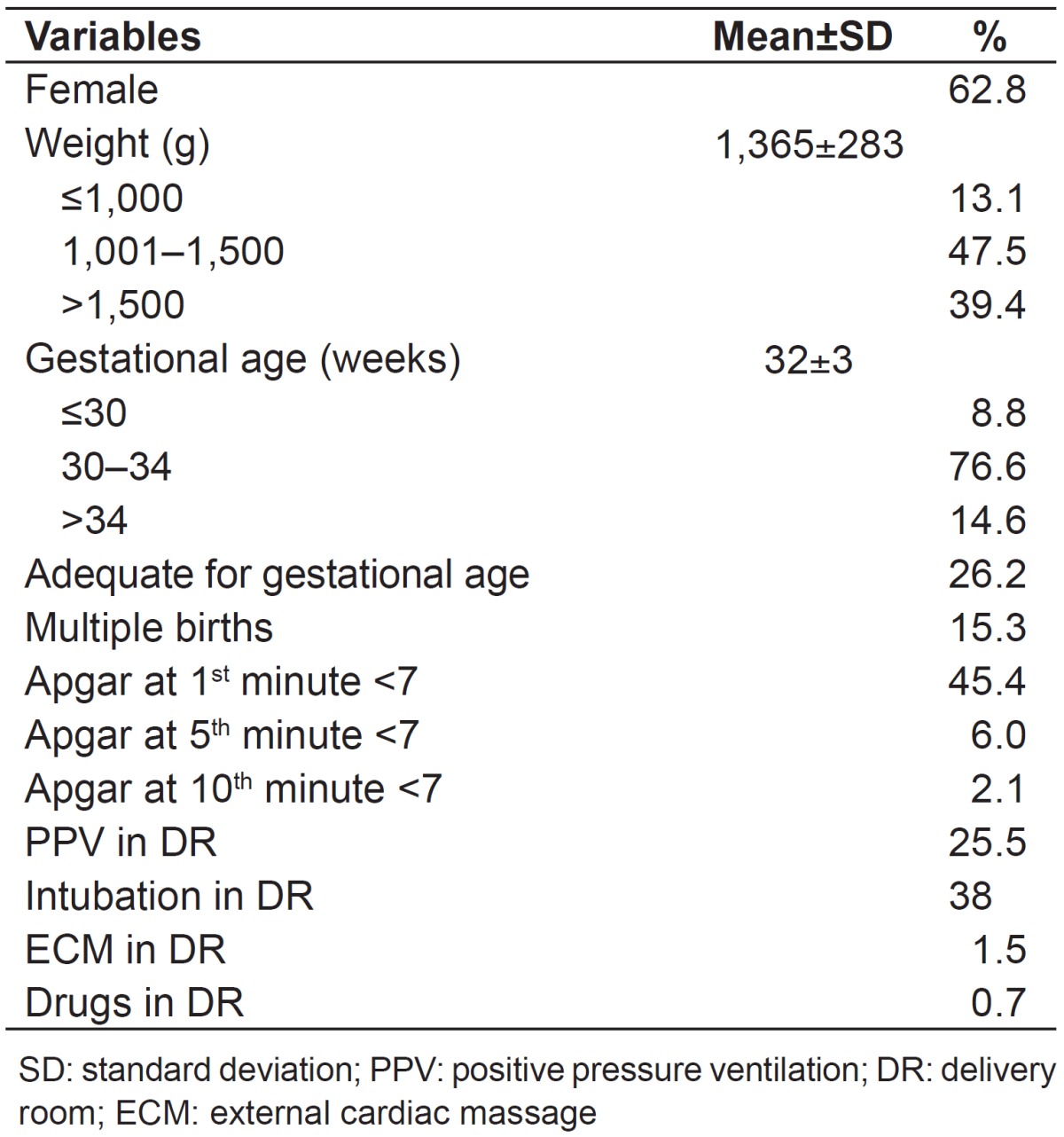
Conditions of birth and care of newborns in the delivery room (n=137)

During stay at the NICU, the most common morbidities were: hypothermia (90.5%), hypoglycemia (34.6%), pneumonia (14.6%), apnea (14.6%), late sepsis (13.9%), and bronchopulmonary dysplasia (13.1%). In 86.1% of participants, at least one transfontanel ultrasonography was performed, with 25.5% showing some degree of hemorrhage. The screening for ROP, according to the service's protocol, was performed at 6 weeks of life in NBs with birth weight <1,500g, what corresponds to 32.8% of the studied sample. There were changes compatible with ROP in 12.4% of screened newborns. The intra-hospital surgery was performed in 2.2% of participants. 

In 52.6% of cases, NBs needed mechanical ventilation and, in 18.2%, there was need for nasal non-invasive ventilation support (CPAP). In 75% of cases, the mechanical ventilation was kept for 4 days, at most. The duration of supplementary oxygen therapy by half the sample was greater than 3 days. The surfactant was administered in 51.8% of PTNBs and, in 75%, it was used in the first 20 minutes of life. A percentage of 16.8% of the sample received vasoactive drug, while 8.8% required third generation antibiotics.

At birth, the mean weight was of 1,365±283g and 26.3% were considered AGA (95%CI 19.1-34.5). In the first 3 days of life, 109 participants (79.5%) began the enteral diet. Maximum weight loss occurred at the 5^th ^day of life. Table 2 shows the weight evolution and the need for nutritional support. 

**Table 2:**
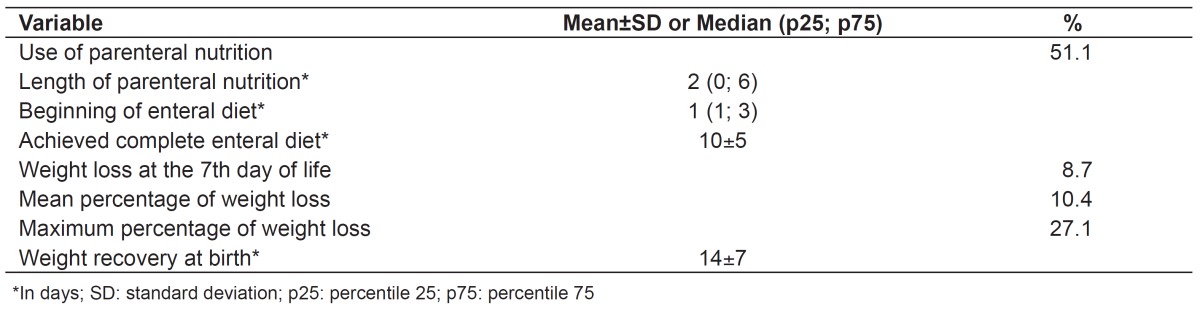
Weight development and need for nutritional support of newborns (n=137)

Patients were admitted at the Kangaroo Nursery with median of 13 days of life (percentile 25=7; percentile 75=28), with mean weight of 1,430±167g. In this moment, 57.7% (95%CI 48.9-66.1) were small for corrected age regarding the Xavier curves^(^ 16 ^)^.

The main intercurrences at the Kangaroo Ward were: anemia (44.5%), apnea (7.4%), and return to the ICU or NICU due to some clinical intercurrence (6.6%). The mean stay was 17±8 days at the Kangaroo Nursery (interval of 3 to 48 days).

Hospital discharge occurred with 36.8±21.8 days of life, with mean weight of 1,780±165g and mean post-concepcional age of 37±5 weeks, being 67.9% (95%CI 59.4-75,6) classified as small for corrected age, according to the Xavier curve^(^ 16 ^)^. Infants who were on breastfeeding were 94.9%, with exclusive breastfeeding in 56.2% of the sample.

Information at 6 months was obtained from 99 children: 76 during follow-up visit, 21 by telephone (type of breastfeeding, readmissions in the period, and performance of corrective surgery of ROP after hospital discharge) and two through hospital records. Of the 99 infants, 16.2% were readmitted within the first 6 months of life and 3% died. Thus, 76 children (55.5% of the sample) were present at the return visit, in average with 186 days of chronological age and 4.5 months of corrected age. In average they weighed 5,954±971g, and 68.4% (95%CI 56.7-78.6) were between the percentiles 15 and 85, according to the WHO curves^(^ 17 ^)^. In total, 40.7% of children were breastfed, with exclusive breastfeeding in 14.4% (95%CI 8.1-23). Table 3 shows data on the NBs at hospital discharge and at 6 months old. The progress and the weight gain adequacy in the different moments analyzed are presented in Table 4.

**Table 3:**
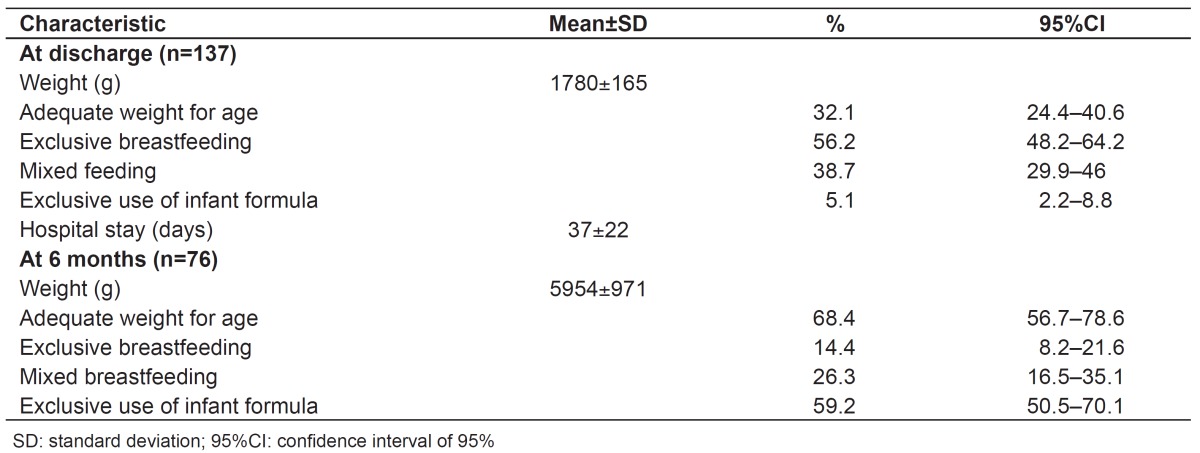
Characteristics of newborns included in the study, at hospital discharge and at 6 months old

**Table 4:**
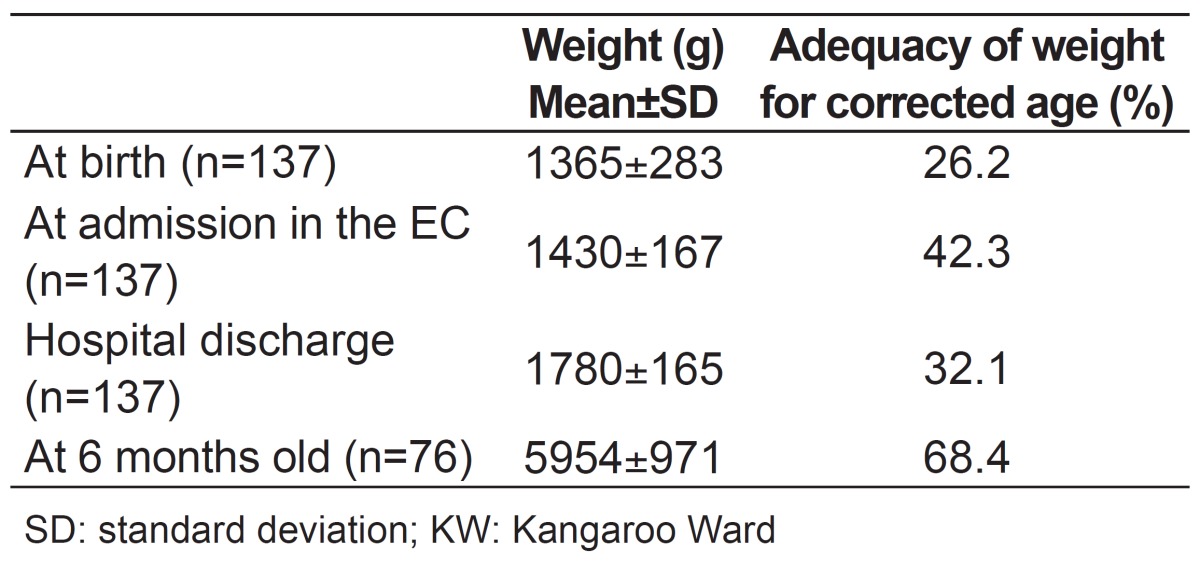
Evolution and weight adequacy of newborns throughout the study, according to corrected age

## Discussion

Several experiences in Brazil and worldwide describe the results of the KM in the care of preterm newborns. Humanization in the assistance improves mother-infant bonding and is perhaps the biggest goal of this practice, seeking the earlier establishment of the relationship and decreasing the risk of abandonment and abuse^(^ 18 ^)^. 

In this study, participants were young mothers, and one quarter of them, adolescents - similar to data described nationally in 2010^(^ 19 ^)^. Most were in a stable relationship, with low monthly income, did not live in the capital, and attended fewer than the 6 prenatal visits recommended by the Ministry of Health. The most prevalent maternal diseases were those that induced premature birth^(^ 20 ^)^. The mean gestational age of the sample was similar to that described by other authors^(^ 21 ^)^ in a study that assessed the results of the KM in Brazil, while the length of stay in the NICU was much lower in the present study, and the length of stay at the Kangaroo Ward was similar.

At birth, we classified most newborns in this study (73.8%) as SGA, demonstrating that there was already a nutritional problem in the intrauterine period. This finding is similar to that reported in a previous study (69%)^(22) ^and diverges from the two other studies^(^ 23 ^,^ 24 ^)^, in which there was a predominance of AGA infants. The characteristics of the population of pregnant women in the maternity where the study was conducted - with morbidities that characterize high risk pregnancies in almost half of them, (such as pregnancy-specific hypertensive disorder, which is a predisposing factor for the occurrence of intrauterine growth restriction^(^ 25 ^))^, besides the low income declared by almost two thirds, hampering the access to nutrients in adequate amounts^(^ 22 ^)^ - may explain this high percentage of SGA newborns, event associated to greater neonatal mortality^(^ 25 ^)^ and greater risk of being malnourished at term^(^ 23 ^)^. Children classified as SGA demonstrated greater growth delay in early life, but there may be late recovery^(^ 26 ^)^, justifying clinical observation during all growth period for early diagnosis and intervention.

At hospital discharge, most infants were classified as small for corrected age, similarly to what was found in the previous study, in which there was a worse weight Z score between birth and 40 weeks, reinforcing that the restriction of post-natal growth is a severe problem in preterm children^(^ 24 ^)^. Other authors^(^ 23 ^)^ described 63.5% of prevalence of malnutrition at term, similar value to that found in the present study. 

A study comparing the evolution of newborns hospitalized in conventional intermediate care units and those admitted to Kangaroo units^(^ 21 ^)^ showed greater weight, height, and head circumference with 36 weeks of corrected age in conventional units, attributing this difference to the nutritional support at the NICU. The factors that determine the growth of preterm infants are still poorly understood, but there is data showing that it remains considerably insufficient^(^ 23 ^)^. Despite being one of the mains pillars of the KM, the frequency of exclusive breastfeeding at hospital discharge in the present study was below that found by other authors who analyzed PTNBs assisted with the KM^(^ 9 ^,^ 21 ^,^ 27 ^)^. Perhaps the low frequency of this practice has contributed to the rate of preterm infants classified as small for corrected age at hospital discharge. 

The effects of postnatal growth restriction (absence or late catch up) are still unknown, including its role in the nutritional adequacy at school age, in the adolescent and the adult, and in the genesis of chronic diseases such as obesity, cardiovascular disease and diabetes, besides lack of proper cognitive development^(^ 28 ^)^.

At 6 months, there was a predominance of infants with adequate weight for corrected age, once two thirds were between the 15th and 85th percentiles of the WHO curve^(^ 17 ^)^, which is equivalent to 2 standard deviations around the mean. This recovery throughout the 1st year of life was also observed among PTNBs assisted by the KM in Colombia^(^ 29 ^)^. 

The time required to reach full diet (10±5 days) was similar to that reported by other authors when analyzing 200 VLBW infants assisted by the conventional method. In this same survey, the variable "presence of full enteral diet by the 10^th^ day of life" appeared as a protective factor for malnutrition at term^(^ 23 ^)^. 

The results obtained in this study, evaluating hospital length of stay and need to return to the de NICU or the ICU, were similar to those obtained in previous studies^(^ 21 ^,^ 27 ^)^, suggesting that the KM is safe, even in overcrowded units and with little human and financial resources, as in the case where the study was conducted. The frequency of apnea, higher than that of a previous study^(^ 21 ^)^ also performed in Brazil, points to the need for more accurate monitoring and appropriate guidance to mothers at the Kangaroo Ward.

The frequency of exclusive breastfeeding in this study at the time of hospital discharge was lower than the reported by authors who analyzed LW infants assisted by the KM^(^ 9 ^,^ 21 ^,^ 27 ^)^. At 6 months, the frequency observed (14.4%) was similar to that described by other authors (22.7%)^(^ 9 ^)^. The low frequency of exclusive breastfeeding found may reflect regional and national characteristics. In a survey conducted in 2008 in Brazilian capitals and the Federal District, including preterm NBs with adequate weight, Aracaju had a median duration of maternal exclusive breastfeeding in younger than 6 months of 49 days, similar to that found in Recife, but lower to that described in Teresina and João Pessoa, which was of 61 days, being the national median 54 days^(^ 30 ^)^. The low rate of exclusive breastfeeding at hospital discharge and at 6 months in children assisted by the KM indicated that interventions are necessary to stimulate this practice by mothers of PTNBs, as well as to improve the understanding of the reasons why the amount of NBs in exclusive breastfeeding falls so intensely after hospital discharge.

During outpatient follow-up, in the first 6 months of life, there was a death rate of 3%, similar to that obtained in a study conducted in Colombia (6%)^(^ 25 ^)^. Information on the causes of the deaths could not be acquired.

A limitation of this study was the lack of a control group, which was due to the absolute majority of mothers willing to join the KM, besides the existence of a number of beds that serves the demand of the place where the study was conducted. The combination of these two factors did not allow the formation of a comparable control group. Other limitations were time of follow-up after discharge and percentage of losses in the consultation at 6 months of life. These factors prevented the application of a multiple regression model to assess associated factors, indicating the need for further studies to complement the present findings.

From the results obtained, it was possible to assess the growth dynamics of preterm infants assisted by the KM at a reference maternity within the Brazilian public unified health system in Northeastern Brazil. The findings show that, in the studied sample, the KM did not interfere negatively on the PTNBs' growth, increasing its potential for use in Brazilian nurseries; on the other hand, they indicate the need for new studies comparing samples of NBs treated by the two different methods, the conventional and the Kangaroo. Therefore, the results presented may be used as a parameter for new studies on the topic, addressing other aspects regarding the preterm's complex process of adaptation to extrauterine life.
