# Supplementary material for: Preterm newborns at Kangaroo Mother Care: a cohort follow-up from birth to six months
Source: Rev Paul Pediatr. 2014 Jun;32(2):171–7. doi: 10.1590/0103-0582201432213113 (PMC4183013; doi:10.1590/0103-0582201432213113)
Supplement: Translation

# Translation: Recém-nascidos prematuros assistidos pelo Método Canguru: avaliação de uma coorte do nascimento aos seis meses

**Authors:** Maria Alexsandra da S. Menezes, Daniela Cavalcante Garcia, Enaldo Vieira de Melo, Rosana Cipolotti

**Affiliations:** 1 UFS, SE, Brasil

**Keywords:** Método Mãe-Canguru, prematuro, crescimento, aleitamento materno

## Abstract

**OBJETIVO:**

Avaliar a evolução clínica, o crescimento e a taxa de aleitamento materno exclusivo de recém-nascidos prematuros assistidos pelo Método Canguru, ao nascimento, na alta e aos seis meses de idade.

**MÉTODOS:**

Estudo prospectivo de uma coorte de prematuros atendidos pelo Método Canguru, em uma maternidade pública de nível terciário do Nordeste do Brasil, com peso ao nascer ≤1750g e em condições clínicas necessárias para se aplicar o método.

**RESULTADOS:**

A amostra constituiu-se de 137 recém-nascidos prematuros, sendo 62,8% do sexo feminino, com peso médio ao nascer de 1365±283g, idade gestacional média de 32±3 semanas e 26,2% eram adequados para a idade gestacional. Foram admitidos na Enfermaria Canguru com mediana de 13 dias de vida, pesando 1430±167g, sendo que, nesse momento, 57,7% foram classificados como pequeno, para a idade gestacional corrigida. Tiveram alta hospitalar com 36,8±21,8 dias de vida, pesando 1780±165g e 67,9% eram pequenos para a idade gestacional corrigida. Aos seis meses de idade cronológica (n=76), tinham peso médio de 5954±971g, estando 68,4% com peso corrigido para a idade gestacional entre os percentis 15 e 85 da curva da Organização Mundial da Saúde (OMS). A frequência de aleitamento materno exclusivo na alta foi de 56,2% e, aos seis meses de idade cronológica, de 14,4%.

**CONCLUSÕES:**

Na amostra estudada, aproximadamente dois terços das crianças assistidas pelo Método Canguru encontravam-se, aos seis meses de idade cronológica, entre os percentis 15 e 85 da curva de peso corporal da OMS. A frequência de aleitamento exclusivo aos seis meses foi baixa.

## Introdução

Há uma estimativa anual de que nascem cerca de 15 milhões de bebês prematuros no mundo, representando mais de um em cada 10 nascimentos. Muitos dos sobreviventes terão problemas de aprendizagem, visuais e/ou auditivos, entre outros^(^ ^1^ ^)^. 

O Programa Mãe-Canguru ou Método Canguru (MC), com origem na Colômbia em 1978, inclui o contato pele a pele entre a mãe e seu recém-nascido (RN) iniciado o mais cedo possível, tendo surgido naquele país como uma alternativa para estimular a alta hospitalar precoce em recém-nascidos de baixo peso (RNBP) estáveis clinicamente^(^ ^2^ ^)^. No Brasil, esse programa tem como objetivo principal incentivar a formação do vínculo entre pais e bebês, sendo uma política nacional de saúde, lançada pelo Ministério da Saúde como Normas de Atenção Humanizada ao Recém-Nascido de Baixo Peso - Método Canguru (NAHRNBP-MC), desde 2000^(^ ^3^ ^)^.

Os principais benefícios atribuídos ao MC incluem: redução de hipotermia, sepse, permanência hospitalar e risco de mortalidade na alta ou com 40 semanas de idade corrigida^(^ ^4^ ^)^, além de impacto positivo no desenvolvimento cognitivo e motor de prematuros^(^ ^5^ ^)^, manutenção da estabilidade durante transporte de prematuros ou recém-nascidos a termo^(^ ^6^ ^)^, bem como dos sinais vitais em níveis fisiológicos, mesmo quando realizado em prematuros sob ventilação mecânica e hemodinamicamente estáveis^(^ ^7^ ^)^. O método evita a separação prolongada entre a mãe e seu bebê, que poderia contribuir para produção insuficiente de leite, baixo vínculo afetivo e aumento de morbidades^(^ ^8^ ^)^, facilitando a amamentação exclusiva para RNBP até o sexto mês de vida^(^ ^9^ ^)^, além de associar-se a maior duração de aleitamento materno e maior produção de leite^(^ ^10^ ^)^. Em recente revisão sistemática publicada pela Cochrane, os autores concluíram que há evidências suficientes para recomendar o uso do MC em RNBP estáveis^(^ ^4^ ^)^. 

Apesar disso, estudo avaliando 176 hospitais-maternidades brasileiros, capacitados entre 2000 e 2003, evidencia que apenas 47,3% implantaram as três etapas do método^(^ ^11^ ^)^. Véras e Traverso-Yépez^(^ ^12^ ^)^ afirmam que a política de implantação do MC não valoriza os aspectos sociais, culturais e ambientais envolvidos na maternagem. Outros autores sugerem que o sucesso da implantação do MC depende do treinamento dos profissionais de saúde e das facilidades e cortesias que o hospital oferece às mães para sua permanência. Além disso, as normas estaduais de implantação, como as ministeriais, não preveem recursos para o seguimento, a monitorização e a avaliação do programa. Nesse processo, a resistência dos profissionais à participação da família tem dado ao MC um caráter hospitalar, com o prolongamento das internações em alojamento conjunto e alta próxima aos critérios convencionais, havendo divergência entre o discurso e a prática^(^ ^13^ ^)^.

Considerando-se essas premissas, o presente estudo teve como objetivo avaliar a evolução clínica, o crescimento e a taxa de aleitamento materno exclusivo de recém-nascidos prematuros assistidos pelo MC em instituição pública da região Nordeste, ao nascimento, na alta e aos seis meses de idade. 

## Método

Estudo de uma coorte prospectiva, realizado em maternidade pública de nível terciário da região Nordeste do Brasil. Nessa unidade, acontecem cerca de 4.400 partos por ano, resultando em 31% de RNBP e 8% de recém-nascidos de muito baixo peso (RNMBP). O serviço dispõe de 36 leitos de Unidade de Terapia Intensiva Neonatal (UTIN), 25 leitos de Unidade de Cuidados Intermediários (UCI), 14 leitos de Enfermaria Canguru e 35 leitos de Alojamento Conjunto, com taxas de ocupação média anual de 121%. A taxa de ocupação da UTIN é frequentemente superior à capacidade do serviço, recorrendo-se, então, a leitos improvisados.

Incluíram-se recém-nascidos prematuros (RNPT) nascidos entre 1º de julho de 2011 e 31 de janeiro de 2012, com peso ao nascer (PN) menor ou igual a 1750g, que estavam em ar ambiente, sem suporte hídrico venoso, pesando mais de 1250g e cuja genitora tenha concordado em participar do MC. Excluíram-se aqueles com malformações congênitas que interferissem na evolução do paciente. 

Pesquisaram-se as seguintes intercorrências clínicas durante a permanência hospitalar: displasia broncopulmonar (DBP), definida como necessidade de oxigenoterapia por 28 dias ou mais após o nascimento; apneia, definida como a cessação de fluxo em vias aéreas superiores com evolução para bradicardia e/ou cianose; hipotermia, definida como temperatura axilar <36,5°C e necessidade de antibióticos de terceira linha (cefalosporina de terceira geração ou vancomicina, isoladas ou em associação).

Obtiveram-se os dados a partir da análise de prontuário, da entrevista com as mães e do exame físico do RN, realizados sempre pelo mesmo examinador. Algumas informações sobre o tipo de aleitamento, correção cirúrgica de retinopatia da prematuridade (ROP) após a alta hospitalar e necessidade de nova internação hospitalar foram obtidas também por telefone, quando a consulta aos seis meses de idade não foi possível. Para se determinar a idade gestacional ao nascer, aplicou-se o Escore New Ballard^(^ ^14^ ^)^ e, para se classificar a adequação ponderal ao nascer, aplicaram-se as curvas de Alexander^(^ ^15^ ^)^, considerando-se adequados para a idade gestacional (AIG) aqueles entre os percentis 10 e 90 e pequenos para a idade gestacional (PIG) os que ficaram abaixo do percentil 10. Para verificar a adequação ponderal à idade corrigida, aplicaram-se as curvas de Xavier^(16) ^à admissão na Enfermaria Canguru e à alta hospitalar. Aos seis meses de idade cronológica e de acordo com a idade corrigida, aplicaram-se as curvas da Organização Mundial da Saúde (OMS)^(^ ^17^ ^)^. 

O local de estudo dispõe de protocolo próprio de seguimento para esses prematuros, mas, para a coleta de dados da pesquisa, agendou-se uma consulta adicional aos seis meses de idade cronológica.

A evolução e a avaliação de adequação ponderal dos RNPT foram realizadas em quatro diferentes momentos: T_0_, ao nascer; T_1_, à admissão na Enfermaria Canguru; T_2_, na alta hospitalar; e T_3_, aos seis meses de idade.

As variáveis categóricas foram apresentadas por meio de frequência, em porcentagem, considerando-se o intervalo de confiança de 95%. A média e o desvio padrão foram calculados para as variáveis quantitativas. Em alguns casos, apresentaram-se tais variáveis em mediana e percentis. Nas medidas de associações entre variáveis qualitativas, utilizaram-se o teste exato de Fisher e do qui-quadrado. Realizou-se a análise estatística por meio do software *Statistical Package for the Social Sciences* (SPSS), versão 13.0 para Windows.

O projeto foi aprovado pelo Comitê de Ética em Pesquisa Envolvendo Seres Humanos da Universidade Federal de Sergipe.

## Resultados

No período de estudo, nasceram 223 RN potencialmente elegíveis, porém 86 foram excluídos (41 por óbito antes da admissão na Enfermaria Canguru, 41 por não adesão da mãe ao método, um por óbito materno e três por recusa materna em participar da pesquisa), sendo a amostra formada por 137 RNPT.

A média de idade materna foi de 26±7 anos, variando de 13 a 44 anos. Mães com até 19 anos constituíram 24,1% da amostra. Conviviam com o pai da criança 81,8%; 66% declararam ter renda familiar mensal de até um salário mínimo e um terço declarou exercer alguma ocupação remunerada; 60,5% procediam do interior de Sergipe ou de outro estado; 44,5% compareceram a quatro ou menos consultas de pré-natal; 41,6% eram primigestas. Entre as mães dos neonatos avaliados, 44,5% tinham histórico de doença hipertensiva específica da gravidez, 36,5% de rotura prematura de membranas e 39% de infecção do trato urinário; 76,6% receberam pelo menos uma dose de corticoide antenatal e 61,3% evoluíram para parto cesáreo.

Quanto ao gênero, 86 RN (62,8%) eram do sexo feminino. Gemelaridade foi encontrada em 15,3% dos participantes. A idade gestacional média ao nascer foi de 32±3 semanas. Os dados sobre as condições do nascimento e assistência aos RNPT em sala de parto estão na Tabela 1. 

**Tabela 1:** Condições do nascimento e assistência aos recém-nascidos em sala de parto (n=137)

Durante o tempo de permanência na UTIN, as morbidades mais comuns foram: hipotermia (90,5%), hipoglicemia (34,6%), pneumonia (14,6%), apneia (14,6%), sepse tardia (13,9%) e displasia broncopulmonar (13,1%). Em 86,1% dos participantes, realizou-se pelo menos uma ultrassonografia transfontanelar, com 25,5% desses mostrando algum grau de hemorragia. A triagem para ROP, conforme protocolo do serviço, foi realizada com seis semanas de vida nos RN com peso ao nascer <1500g, o que corresponde a 32,8% da amostra estudada. Houve alterações compatíveis com ROP em 12,4% dos neonatos triados. A cirurgia intra-hospitalar foi realizada em 2,2% dos participantes. 

Em 52,6% dos casos, o RN necessitou de ventilação mecânica e, em 18,2%, de modalidade de suporte ventilatório não invasivo (CPAP) nasal. Em 75% dos casos, a ventilação mecânica foi mantida por, no máximo, quatro dias. A duração de oxigenoterapia suplementar por metade da amostra foi superior a três dias. O surfactante foi administrado em 51,8% dos RNPT e, em 75%, foi utilizado nos primeiros 20 minutos de vida. Receberam droga vasoativa 16,8% da amostra, enquanto 8,8% necessitaram usar antibiótico de terceira linha.

Ao nascer, o peso médio foi de 1365±283g e 26,3% foram considerados AIG (IC95% 19,1-34,5). Nos três primeiros dias de vida, 109 participantes do estudo (79,5%) iniciaram a dieta enteral. A perda ponderal máxima ocorreu no quinto dia de vida. A Tabela 2 mostra a evolução ponderal e as necessidades de suporte nutricional. 

**Tabela 2:** Evolução ponderal e necessidade de suporte nutricional dos recém-nascidos (n=137)

Os pacientes foram admitidos na Enfermaria Canguru com mediana de 13 dias de vida (percentil 25=7; percentil 75=28), com peso médio de 1430±167g. Nesse momento, 57,7% (IC95% 48,9-66,1) eram pequenos para a idade corrigida, segundo as curvas de Xavier^(^ ^16^ ^)^.

Os principais eventos ocorridos na Enfermaria Canguru foram: anemia (44,5%), apneia (7,4%) e retorno para UCI ou UTIN por alguma complicação clínica (6,6%). A permanência média foi de 17±8 dias na Enfermaria Canguru (intervalo de três a 48 dias).

A alta hospitalar ocorreu com 36,8±21,8 dias de vida, média de peso de 1780±165g e de idade pós-concepcional de 37±5 semanas, sendo 67,9% (IC95% 59,4-75,6) classificados como pequenos para a idade corrigida, conforme a curva de Xavier^(^ ^16^ ^)^. Estavam em aleitamento materno 94,9%, sendo exclusivo em 56,2% da amostra.

Informações aos seis meses foram obtidas de 99 crianças: 76 durante consulta de seguimento, 21 por contato telefônico (tipo de aleitamento materno, reinternações no período e realização de cirurgia corretiva de ROP após a alta hospitalar) e duas por meio dos registros hospitalares. Dos 99 lactentes, 16,2% foram reinternados nos primeiros seis meses de vida e 3% foram a óbito. Assim, à consulta de retorno, em média com 186 dias de idade cronológica e 4,5 meses de idade gestacional corrigida, compareceram 76 crianças, equivalendo a 55,5% da amostra. Pesaram, em média, 5954±971g, estando 68,4% (IC95% 56,7-78,6) entre os percentis 15 e 85, segundo as curvas da OMS^(^ ^17^ ^)^. Estavam em aleitamento materno 40,7% das crianças, sendo exclusivo em 14,4% (IC95% 8,1-23). A Tabela 3 contém dados sobre os RN na alta hospitalar e aos seis meses de idade. A evolução e a adequação ponderal dos RN nos diferentes momentos analisados são apresentadas na Tabela 4.

**Tabela 3:** Características dos recém-nascidos incluídos no estudo, na alta hospitalar e aos seis meses de idade

**Tabela 4:** Evolução e adequação ponderal dos recém-nascidos ao longo do estudo, conforme idade corrigida

## Discussão

Várias experiências no Brasil e no mundo descrevem os resultados do MC no atendimento ao RNPT. A humanização à assistência melhora o vínculo mãe-filho e talvez seja o maior objetivo dessa prática, buscando o estabelecimento mais precoce da relação e diminuindo o risco de abandono e de maus-tratos^(^ ^18^ ^)^. 

Neste estudo, as mães participantes eram jovens, sendo um quarto adolescentes - dado semelhante ao descrito em âmbito nacional em 2010^(^ ^19^ ^)^. A maioria estava em um relacionamento estável, com renda mensal baixa, não residia na capital e frequentou menos que as seis consultas de pré-natal preconizadas pelo Ministério da Saúde. As doenças maternas mais prevalentes foram aquelas implicadas como favorecedoras de um parto prematuro^(^ ^20^ ^)^. A idade gestacional média da amostra foi próxima à descrita por outros autores^(^ ^21^ ^)^ em pesquisa que avaliou os resultados do MC no Brasil, sendo o tempo de permanência em UTIN bem menor do que o descrito nessa mesma pesquisa, enquanto o tempo de permanência em Enfermaria Canguru foi semelhante.

Ao nascer, classificou-se a maior parte dos RN desta pesquisa (73,8%) como PIG, demonstrando que já havia agravo nutricional no período intrauterino. Esse achado é próximo ao observado em estudo anterior (69%)^(22) ^e discorda de outros dois estudos^(^ ^23^ ^,^ ^24^ ^)^, nos quais havia predomínio de bebês AIG. As características da população de gestantes atendidas na maternidade onde o estudo foi realizado - com morbidades que caracterizam gestações de alto risco em quase metade delas, como a doença hipertensiva específica da gestação, que é fator predisponente para a restrição de crescimento intrauterino^(^ ^25^ ^)^, além da baixa renda declarada por quase dois terços, prejudicando o acesso a nutrientes em quantidades adequadas^(^ ^22^ ^)^ - podem explicar esse percentual elevado de RN PIG, evento associado à maior mortalidade neonatal^(^ ^25^ ^)^ e ao maior risco de ser desnutrido ao termo^(^ ^23^ ^)^. Crianças classificadas como PIG mostram maior atraso do crescimento nos primeiros anos de vida, porém pode haver recuperação tardia^(^ ^26^ ^)^, justificando a vigilância clínica durante todo o período de crescimento para diagnóstico e intervenção precoces.

À alta hospitalar, a maioria dos bebês foi classificada como pequena para a idade corrigida, de modo similar ao que foi encontrado em estudo anterior, no qual houve piora do escore Z de peso entre o nascimento e 40 semanas, reforçando que a restrição do crescimento pós-natal é um problema grave nos prematuros^(^ ^24^ ^)^. Outros autores^(^ ^23^ ^)^ descreveram 63,5% de prevalência de desnutrição ao termo, valor bem semelhante ao da presente pesquisa. 

Estudo comparando a evolução de recém-nascidos internados em unidades intermediárias convencionais com aqueles internados em unidades canguru^(^ ^21^ ^)^ evidenciou maior peso, estatura e perímetro cefálico com 36 semanas de idade corrigida nas unidades convencionais, atribuindo tal diferença ao suporte nutricional realizado na UTI. Os fatores que determinam o crescimento de prematuros ainda são pouco conhecidos, mas há dados que constatam que o mesmo continua consideravelmente insuficiente^(^ ^23^ ^)^. Mesmo sendo o incentivo ao aleitamento materno um dos principais pilares do MC, sua frequência na forma exclusiva à alta hospitalar encontrada no presente estudo esteve abaixo da encontrada por outros autores que analisaram RNBP assistidos pelo MC^(^ ^9^ ^,^ ^21^ ^,^ ^27^ ^)^. Talvez a baixa frequência dessa prática tenha contribuído para o índice de RNPT classificados como pequenos para a idade corrigida à alta hospitalar. 

Os efeitos da restrição do crescimento (ausência ou atraso no *catch up*) pós-natal ainda não são suficientemente conhecidos, inclusive seu papel na adequação nutricional na idade escolar, no adolescente e no adulto e na gênese de doenças crônicas como obesidade, doença cardiovascular e diabetes, além de falta de desenvolvimento cognitivo adequado^(^ ^28^ ^)^.

Aos seis meses, houve predomínio de bebês com peso adequado para a idade corrigida, uma vez que dois terços encontravam-se entre os percentis 15 e 85 da curva da OMS^(^ ^17^ ^)^, o que equivale a dois desvios padrão em torno da média. Essa recuperação ao longo do primeiro ano de vida também foi observada entre RNPT assistidos pelo MC na Colômbia^(^ ^29^ ^)^. 

O tempo necessário para atingir a dieta plena (10±5 dias) foi semelhante ao relatado por outros autores ao analisarem 200 RNMBP assistidos pelo método convencional. Nessa mesma pesquisa, a variável "presença de dieta enteral plena até o 10º dia de vida" apresentou-se como fator de proteção para a desnutrição ao termo^(^ ^23^ ^)^. 

Os resultados obtidos no presente estudo, ao se avaliarem tempo de hospitalização e necessidade de retorno à UTIN ou à UCI, assemelham-se aos obtidos em estudos anteriores^(^ ^21^ ^,^ ^27^ ^)^, sugerindo ser o MC seguro, mesmo em unidades com superlotação e com poucos recursos financeiros e humanos, como é o caso do local onde o estudo foi realizado. A frequência de apneia, superior à de estudo anterior^(^ ^21^ ^)^ também realizado no Brasil, aponta para a necessidade de monitoramento mais acurado e orientação adequada às mães em Enfermaria Canguru.

A frequência de aleitamento materno exclusivo encontrada neste estudo por ocasião da alta hospitalar foi inferior à referida por outros autores que analisaram RNBP assistidos pelo MC^(^ ^9^ ^,^ ^21^ ^,^ ^27^ ^)^. Aos seis meses, a frequência observada (14,4%) foi semelhante à descrita por outros autores (22,7%)^(^ ^9^ ^)^. A baixa frequência de amamentação materna exclusiva encontrada pode refletir características regionais e nacionais. Em pesquisa realizada em 2008 nas capitais brasileiras e no Distrito Federal, incluindo RN de termo e com peso adequado, Aracaju teve duração mediana de aleitamento materno exclusivo em menores de seis meses de 49 dias, semelhante à encontrada em Recife, porém inferior à descrita em Teresina e João Pessoa, que foi de 61 dias, sendo a mediana nacional de 54 dias^(^ ^30^ ^)^. A baixa frequência de aleitamento materno exclusivo na alta e aos seis meses em crianças assistidas pelo MC indica que são necessárias intervenções para estimular essa prática por mães de RNPT, assim como melhorar a compreensão dos motivos pelos quais a proporção de RN em aleitamento materno exclusivo decai tão intensamente após a alta hospitalar.

Durante o seguimento ambulatorial, nos primeiros seis meses de vida, constatou-se taxa de óbitos de 3%, semelhante à obtida em estudo realizado na Colômbia (6%)^(^ ^25^ ^)^. Informações sobre as causas dos óbitos não puderam ser adquiridas.

Uma limitação deste estudo foi a falta de um grupo controle, o que aconteceu pelo fato de, na maternidade estudada, a absoluta maioria das mães desejar antecipadamente participar do MC, além de haver um número de leitos que atende à demanda do local de estudo. A combinação desses dois fatores não permitiu a formação de um grupo controle comparável. Outras limitações foram o tempo de seguimento após a alta e o percentual de perdas na consulta aos seis meses de vida. Esses fatores inviabilizaram a aplicação de um modelo de regressão múltipla para se avaliarem os fatores associados, indicando a necessidade de novos estudos que complementem os presentes achados.

A partir dos resultados obtidos, foi possível conhecer a dinâmica de crescimento dos RNPT assistidos pelo MC em uma maternidade de referência para o Sistema Único de Saúde na região Nordeste do Brasil. Os achados sugerem que, na amostra estudada, o MC não interferiu negativamente no crescimento de RNPT, reforçando seu potencial de utilização em maternidades brasileiras; por outro lado, indica a necessidade de novos estudos comparando-se amostras de RN assistidos pelos diferentes métodos, convencional e Canguru. Dessa forma, os resultados apresentados podem ser utilizados como parâmetro de comparação para novas pesquisas sobre o tema, as quais venham a abordar outros aspectos do complexo processo de adaptação do bebê prematuro à vida extrauterina.
